# Pathogenetic mechanisms of heatstroke and novel therapies

**DOI:** 10.1186/cc11265

**Published:** 2012-06-07

**Authors:** Abderrezak Bouchama

**Affiliations:** 1 King Abdullah International Medical Research Center, Riyadh, Kingdom of Saudi Arabia

## 

Heatstroke, a life-threatening condition defined by a rapidly increasing core temperature greater than 40°C and multiple organ system dysfunction, is a leading cause of morbidity and mortality during heat waves [1]. The heat wave that affected Europe during August 2003 led to an unprecedented 70,000 excess deaths of which up to 40% were confirmed to be due to heatstroke [2]. Sophisticated climate models predict increasing frequency and severity of heat waves and so the incidence of heat-related death could rise if proactive measures and novel therapy to address this threat are not adopted [3].

The mechanisms of multiple organ system dysfunction in heatstroke are not fully understood and include direct tissue injury and cell death resulting from heat cytotoxicity together with delayed organ dysfunction and damage secondary to activation of inflammatory and coagulation pathways. Histopathological changes include endothelial injury, disseminated intravascular thrombosis, neutrophils infiltration and apoptosis [4,5]. Despite cooling and optimal treatment in intensive care the overall mortality from heatstroke exceeds 60%, due in large part to the fact that no specific treatment is available [6,7].

Experimental evidence from rodent models of heatstroke suggest that immunomodulation of the host response may alter the clinical course of heatstroke and thereby improve outcome [8-10]. Heatstroke in induces systemic and local (central nervous system) production of TNFα and IL-1, and severe coagulopathy. This is associated with multiple organ system dysfunction including severe neuronal injury, and high mortality. Administration of an IL-1 receptor antagonist [8], corticosteroids [9] or activated protein C [10] before the onset of heatstroke attenuates the multiple organ system dysfunction and improves survival. However, extrapolation of these data from these small animal models to humans is problematic because of inter-species differences.

Baboons represent the most appropriate model for the study of the host inflammatory and hemostatic responses to heat stress and their relation to cellular injury and death as well as for testing novel therapy, which targets these pathways. Moreover, the findings may have direct applicability to human heatstroke, and could represent the basis for clinical trial. In this review, we show that baboons subjected to heat stress reproduce both the clinical and immunological changes similar to those seen in humans [11]. Using this experimental model, we report that immunomodulating the host systemic inflammatory and coagulation responses failed to translate into improved survival, suggesting that further basic research on the pathogenesis of heatstroke is required [12-14].
